# Novel Transethosomal Gel Containing Miconazole Nitrate; Development, Characterization, and Enhanced Antifungal Activity

**DOI:** 10.3390/pharmaceutics15112537

**Published:** 2023-10-27

**Authors:** Zara Asghar, Talha Jamshaid, Muhammad Sajid-ur-Rehman, Usama Jamshaid, Heba A. Gad

**Affiliations:** 1 Department of Pharmaceutics, Faculty of Pharmacy, The Islamia University of Bahawalpur, Bahawalpur 63100, Pakistan; zaraasghar21m@gmail.com (Z.A.); sj_pharmacist@iub.edu.pk (M.S.-u.-R.); 2 Department of Pharmaceutics, Faculty of Pharmacy, Strasbourg University, 67084 Strasbourg, France; usama.jamshaid042@gmail.com; 3 Department of Pharmaceutics and Industrial Pharmacy, Faculty of Pharmacy, Ain Shams University, Cairo 11566, Egypt; 4 Department of Pharmaceutical Sciences, Pharmacy Program, Batterjee Medical College, Jeddah 21442, Saudi Arabia

**Keywords:** in vitro antifungal activity, miconazole nitrate, transethosomes, carbopol 934, gel, ex vivo permeation, skin irritation test, health care

## Abstract

Miconazole nitrate (MCNR) is a BCS class II antifungal drug with poor water solubility. Although numerous attempts have been made to increase its solubility, formulation researchers struggle with this significant issue. Transethosomes are promising novel nanocarriers for improving the solubility and penetration of drugs that are inadequately soluble and permeable. Thus, the objective of this study was to develop MCNR-loaded transethosomal gel in order to enhance skin permeation and antifungal activity. MCNR-loaded transethosomes (MCNR-TEs) were generated using the thin film hydration method and evaluated for their zeta potential, particle size, polydispersity index, and entrapment efficiency (EE%). SEM, FTIR, and DSC analyses were also done to characterize the optimized formulation of MCNR-TEs (MT-8). The optimized formulation of MCNR-TEs was incorporated into a carbopol 934 gel base to form transethosomal gel (MNTG) that was subjected to ex vivo permeation and drug release studies. In vitro antifungal activity was carried out against *Candida albicans* through the cup plate technique. An in vivo skin irritation test was also performed on Wistar albino rats. MT-8 displayed smooth spherical transethosomal nanoparticles with the highest EE% (89.93 ± 1.32%), lowest particle size (139.3 ± 1.14 nm), polydispersity index (0.188 ± 0.05), and zeta potential (−18.1 ± 0.10 mV). The release profile of MT-8 displayed an initial burst followed by sustained release, and the release data were best fitted with the Korsmeyer-Peppas model. MCNR-loaded transethosomal gel was stable and showed a non-Newtonian flow. It was found that ex vivo drug permeation of MNTG was 48.76%, which was significantly higher than that of MNPG (plain gel) (*p* ≤ 0.05) following a 24-h permeation study. The prepared MCNR transethosomal gel exhibited increased antifungal activity, and its safety was proven by the results of an in vivo skin irritation test. Therefore, the developed transethosomal gel can be a proficient drug delivery system via a topical route with enhanced antifungal activity and skin permeability.

## 1. Introduction

Topical drug products represent a noteworthy category of drug delivery systems, and their usage in therapy is becoming more common. Topical dosage forms conveniently distribute drugs to a specific site of injured or infected skin [1]. The prevalence of fungi-related dermatological issues like dermatophytosis and candidiasis is rising dramatically. Successful treatment depends on the drug’s capability to penetrate the skin and be stored in the deeper layers of the skin. However, the therapeutic efficacy of a topically applied product used to treat dermatological conditions is sometimes as widespread as intended [2]. Fungal infections are cutaneous infections that affect the skin, nails, and mucous membranes. Candidiasis is one of the most common superficial fungal infections that can penetrate into deeper tissues when the immune system is compromised. Underarms and the intergluteal regions are frequently affected because they are moist, heated, and creased [3]. Topical treatment for fungal infections is preferred over systemic treatment; as the drug is delivered straight to the infection, there are fewer adverse effects and higher levels of patient compliance [4]. However, the stratum corneum, the top layer of skin, serves as the main barrier against drug penetration. Therefore, it is essential to develop a drug delivery system for antifungal drugs that can get through the stratum corneum’s barriers [4,5]. The capability of the antifungal drugs to pass through the layers of skin and reach the target spot determines their effectiveness [6]. Topical fungal infections are now treated using a variety of conventional formulations, including gels, creams, lotions, and ointments. However, these formulations have limitations, such as poor penetration and retention, that render them ineffective [7].

Miconazole nitrate (MCNR) is a broad-spectrum antifungal drug with an imidazole group used to treat candidiasis. MCNR has a lipophilic nature. It has a weak water solubility (0.1 mg/mL) and a significant hepatic transformation (metabolic changes), contributing to its low systemic effectiveness [8]. It works as an antifungal drug in two distinct manners: by blocking the formation of ergosterol and peroxidases, which causes an internal buildup of peroxide and ultimately cell death [9]. There are many ways to overcome its low water solubility, particularly using lipid nanoparticles. Numerous delivery systems, including solid lipid nanoparticles, organogels, transfersomal gels, transethosomal gels, and nanocapsules, have been found to improve the solubility and permeation of the drug through the skin and enhance the antifungal activity of MCNR [9,10].

Topical administration creates difficulties in treating cutaneous illnesses due to restricted skin penetration. Lipid vesicles as carriers of topical medications have drawn much attention over the past few decades due to their ability to bypass the natural skin barrier [9]. Utilizing nanosized vesicles is a unique method of topical delivery to the skin because they may reach deeper layers of the skin, being adsorbed at the skin borders or changing its barrier properties, allowing molecules to reach the stratum corneum layer. Additionally, due to the lipid bilayer in the formulation, they can also fluidize the stratum corneum and speed up penetration [11]. 

Transethosomes are novel nano-carriers made up of lipid, edge activator, and an alcohol that function in the vesicular system. Recently, the name “trans-ethosomes” was given to lipid-based nano-vesicles that contain an edge activator and ethanol. The benefits of ethosomes and transfersomes are combined in transethosomes. Transethosomes provide a number of advantages over other drug delivery methods because of the inclusion of these components. Ethanol improves drug penetration through the minute holes created in the stratum corneum as a result of fluidization by increasing the fluidity of lipids and decreasing the density of the lipid bilayer [12]. The phospholipid bilayer in these vesicles is weakened by the edge activator, which also renders the vesicle ultradeformable and extremely flexible. When a suitable ratio of an appropriate lipid and edge activator is used, the edge activators (EAs) perform exceptionally well as membrane-destabilizing agents to increase the deformability of vesicle membranes. This mixture allows the transethosomes to become deformable and ultra-flexible, which results in a higher permeability [13]. Due to the fact that this has a significant impact on entrapment efficiencies, vesicle size, and penetration ability, the lipid: edge activator ratio should be optimized [14].

Moreover, addition of vesicular nano-formulations to gel systems improves the therapeutic efficiency by increasing drug permeability and residence time. Carbopol is a commonly used gelling ingredient that is extensively utilized in the fabrication of gels. Carbopol 934 is a polyacrylic polymer that can produce effective viscosity [15]. It has a cross-linked structure and is hydrophilic [16]. It is frequently employed in transdermal distribution as a permeation enhancer and is rate-controlling [17]. 

The current research aimed to develop and characterize MNTG (transethosomal gel) as a novel topical drug delivery system for enhancing antifungal activity and skin permeability of MCNR. Finally, ex vivo permeation, in vitro antifungal activity, and in vivo skin irritation studies were performed on the dorsal skin of rats for the prepared MCNR-loaded transethosomal gel (MNTG) and compared with plain gel and marketed cream (Daktarin^®^ cream 2%) in order to confirm the safety of the developed gel for cutaneous application.

## 2. Materials and Methods

### 2.1. Materials

MCNR was donated by ATCO laboratories Limited, Karachi, Pakistan. Lecithin was a kind gift from Lipoid GmbH, Ludwigshafen, Germany. Oleic acid, chloroform, methanol, ethanol, and triethanolamine were bought from Sigma Aldrich (St. Louis, MO, USA). Carbopol-934 was purchased from Merck KGaA (Darmstadt, Germany). Double distilled water was acquired from the pharmaceutics laboratory of the Islamia University of Bahawalpur, Pakistan. All chemicals used to develop the transethosomal vesicles were of analytical grade. *Candida albicans* (ATCC 10231) was obtained from LABNOSTIX Ltd. (Warrington, UK). 

### 2.2. Preparation of MCNR-Loaded Transethosomes

Several trials were conducted by adjusting the quantities of lipid, oleic acid, and ethanol, as shown in Table 1, in order to establish an optimized transethosomal formulation with characteristics of high entrapment efficiency, ideal vesicle size, polydispersity index, and zeta potential. Oleic acid (OA) was used as an edge activator (EA). Oleic acid was used in combination with lecithin as lecithin: OA. The traditional thin-film hydration method was used to prepare the MCNR-loaded transethosomes (TEs) [5]. In a dry round-bottom flask, MCNR with oleic acid and lecithin were mixed in 3:1 of chloroform and methanol mixture. A thin lipid film was prepared on the wall of the round-bottom flask by allowing the organic solvents to vaporize through a rotary evaporator (Heidolph, Kelheim, Germany) set to 60 rpm, under low pressure at 55 °C. The final solvent residues were eliminated under vacuum for an entire night. Ethanol and phosphate buffer solution (pH 5.5) were then used to hydrate the film for 1 h at 100 revolutions per minute at room temperature. The preparation was then stored at 4 °C for further studies after being sonicated for 15–20 min to minimize the particle size [18]. All formulations were packed in well-sealed plastic containers for further evaluation. 

### 2.3. In Vitro Characterization of MCNR-Loaded Transethosomal Formulations 

#### 2.3.1. Entrapment Efficiency (%EE)

An indirect method was used to calculate the entrapment efficiency (% EE) of transethosomal suspensions [19]. Transethosomal suspensions underwent ultracentrifugation for half an hour at 12,000× *g*, and then clear supernatants were obtained. The collected supernatants were subjected to spectrophotometric analysis for quantification of MCNR concentration in transethosomal formulations. The measurements of total amount of drug and free drug amount were carried out at λ_max_ = 272 nm. A calibration curve was used to determine the drug concentration. The calibration equation of the standard curve was used to measure the free drug amount in the supernatant (R^2^ = 0.998). Readings were obtained in triplicate, and the percentage of EE was calculated as follows:

$$\%EE=\frac{Total\;drug\;amount-Free\;drug\;amount}{Total\;drug\;amount}\times 100$$

#### 2.3.2. Particle size, Polydispersity Index (PDI), and Zeta Potential

The average particle size (PS) and electrostatic potential of charge of suspended transethosomes (TEs), which are crucial characteristics for stability studies, were evaluated [19,20]. Zeta potential (ZP) was used to determine whether the charged particles were repelling or attracting one another. The mean PS, PDI, and ZP of the prepared MCNR-loaded transethosomes were determined using the Zetasizer ZS90 (Malvern, UK). According to the specifications, the samples were diluted with deionized water, and scanning was carried out at 25 °C with an angle of scattering of 90° [21]. 

### 2.4. Optimum Formula Selection

The formulation with the lowest PDI, PS, highest EE%, and ZP was chosen as the optimized formula for further research. The best formula was selected based on the desired factor closest to one.

### 2.5. In Vitro Characterization of Optimum MCNR Formulation

#### 2.5.1. Scanning Electron Microscopy (SEM)

The shape and surface morphology of transethosomes were studied using a scanning electron microscope (JSM-7610FPlus, JEOL, Peabody, MA, USA). One drop of the optimized formulation (MT-8) was put on a transparent glass stub. It was air dried, coated with gold using an E 5100 Sputter coater, and then examined via SEM [22].

#### 2.5.2. Fourier-Transform Infrared Spectroscopy (FT-IR) Analysis

The compatibility of drug with the transethosomal components was tested through infrared (IR) spectroscopy [23]. The IR spectra of MCNR, oleic acid, lecithin, and optimized MCNR-loaded transethosomal formulation (MT-8) were measured using a Fourier-transform infrared spectrophotometer (Bruker, Rheinstetten, Germany). Each sample was put into a disc after being compressed and underwent scanning from 4000 cm^−1^ to 500 cm^−1^ [24].

#### 2.5.3. Differential Scanning Calorimetry (DSC) Analysis

The thermal properties of pure MCNR, lecithin, oleic acid, and the optimized MCNR-loaded transethosomal formulation (MT-8) were examined using a Shimadzu DSC Q-2000 apparatus (Tokyo, Japan). Each sample was weighed precisely and placed in an aluminum pan with a nitrogen environment. The temperature range for the DSC thermograms was 0 to 300 °C, with a scanning rate of 10 °C per minute [25]. 

#### 2.5.4. Stability Studies of Optimized MCNR Formulation

The optimized transethosomal formulation (MT-8), having the highest entrapment efficiency, optimum vesicle size, polydispersity index, and zeta potential, was kept at 4 °C and 25 °C for 90 days. Samples were taken on day 0 and after 45 and 90 days. Stability was assessed by comparing the results of the initial measurements with those obtained after storage. The vesicles’ pH, PS, PDI, and EE% were assessed as previously mentioned. Vesicles were visually inspected for changes in morphology [5].

### 2.6. Preparation of Transethosomal Gel

Transethosomes loaded with MCNR were delivered topically using carbopol 934 as a carrier. Carbopol 934 (1.5% *w*/*w*) was used as a gelling agent to prepare MCNR-loaded transethosomal gel (MNTG). A precisely weighed proportion of carbopol was soaked in distilled water overnight [26]. Drops of triethanolamine were added for the adjustment of pH of gel while swirling continuously in a homogenizer (Euro-Star, IKA D 230, Staufen, Germany), resulting in the formation of a clear, translucent homogenized gel with a pH between 5.5 and 6.5. To develop a homogeneous gel formulation including transethosomal vesicles, optimized MCNR-loaded transethosomal suspension was slowly added to the gel while continually mixed with a homogenizer [27]. The pH of the transethosomal gel (MNTG) and plain gel (MNPG) that had been freshly made were accustomed to within the natural skin pH range (4.5–6.0).

### 2.7. Evaluation of Gels

#### 2.7.1. Organoleptic Investigation and Stability Studies

The organoleptic characteristics of MCNR transethosomal gel (MNTG) and MCNR plain gel (MNPG), such as the gel’s appearance, color, smell, and liquefaction, as well as their relative thermal stability at different storage temperatures e.g., 8 °C, 25 °C, 40 °C, and 40 °C ± 75% RH and at specific time intervals (0, 1, 7, 14, 28, 45, 60, and 90 days) were evaluated for three months (90 days) [27]. 

#### 2.7.2. Analysis of pH

The pH of the transethosomal gel (MNTG) and plain gel (MNPG) that had been freshly made were first accustomed within the natural skin pH range (4.5–6.0). After that, triplicate readings of all the formulations were taken at predetermined intervals and stored at various conditions of temperature and humidity (previously defined) for 90 days [28]. 

#### 2.7.3. Measurement of Conductivity

The conductivity measurements of both freshly developed transethosomal gel (MNTG) and plain gel (MNPG) formulations were taken with the aid of a conductivity meter (WTW COND-197i, Weilheim Germany) at specific time intervals (0, 1, 7, 14, 28, 45, 60, and 90 days) and at different storage temperatures e.g., 8 °C, 25 °C, 40 °C, and 40 °C ± 75% RH for whole study period of 90 days [29]. The conductivity meter probe was placed into the beaker containing the gel sample to measure the electrical conductivity of the gels. The electrical conductivity was recorded three times, and the mean was determined.

#### 2.7.4. Viscosity Analysis

Rheological studies of MNTG and MNPG were performed at a temperature of 25 °C using a programmable Ultra Rheometer DV-III (Brookfield Engineering Laboratories, Middleborough, MA, USA) with spindle number CP41 and Rheocalc version 2.5.6, where the Software Rheocalc V-2.6 was used for examining the data. Studies of viscosity are crucial to evaluate a semisolid system’s stability. MNTG and MNPG were subjected to different temperatures e.g., 8 °C, 25 °C, 40 °C, and 40 °C ± 75% RH for 90 days to examine their thermal stability. The viscosity and shear rate of each sample were measured. The sample of each gel (0.5 ± 0.01 g) was put in a sample holding cup and spun between 20 and 100 revolutions per minute for analysis [29]. 

#### 2.7.5. Spreadability

The spreadability of MNTG and MNPG kept at different storage temperatures, i.e., 8 °C, 25 °C, 40 °C, and 40 °C ± 75% RH was determined using Milla’s approach at specific time intervals (0, 1, 7, 14, 28, 45, 60, and 90 days) for study period of 90 days [30]. Readings were taken in triplicate. On a glass slide, a two-centimeter circle was drawn, and it was filled with 0.5 g of gel. A second glass slide was positioned on top of the first one, and a 500 g of weight was placed on the upper slide. Any change in the circle’s diameter was then measured [29].

### 2.8. In Vitro Release Study and Drug Release Kinetics

An in vitro release study was carried out using a dialysis membrane having molecular weight cutoff value of 12–14,000 Da (Sigma-Aldrich, St. Louis, MO, USA). Release study was performed via the type II dissolution apparatus (Pharma Test, Hainburg, Germany). Dialysis bags were slit, and one end was knotted to prevent leakage. MCNR solution (MCNR-SOL), optimized MCNR transethosomal formulation (MT-8), optimized formulation (MT-8)-loaded transethosomal gel (MNTG), and MCNR solution-loaded plain gel (MNPG) were added into their corresponding dialysis bags and then sealed in order to compare their release pattern. The dialysis bags were immersed in 250 mL buffer solution with pH 5.5 at 37 °C. Samples (5 mL) of the medium were taken out periodically at 0, 0.25, 0.5, 1, 2, 3, 4, 5, 6, 12, and 24 h and replaced with the same volume of fresh medium to keep the sink state constant. The amount of released drug in the buffer was determined using a UV-vis spectrophotometer (Bio-Tek Instruments, Bad Friedrichshall, Germany) at (λ_max_= 272 nm). The analysis was carried out three times, and the average value was recorded.

The kinetic model equations of first-order, zero-order, Higuchi model, and Korsmeyer–Peppas model of the drug release kinetics were used to determine drug release patterns [31,32]. Regarding the Korsmeyer–Peppas model, if “n” is less than or equals 0.45, this is referred to as Fickian diffusion. If “n” is greater than 0.45, it denotes non-Fickian diffusion. However, when “n” is between 0.45 and 0.89, this is referred to as the anomalous system. 

### 2.9. Ex Vivo Permeation

Ex vivo permeation was employed using the Franz cell method, where the MNTG (transethosomal gel) and MNPG (plain gel) were applied on the abdominal skin of Wistar albino rats, which is the most similar to that of human natural skin [33]. First, the rat skin was collected and cleaned with a regular saline solution. After carefully separating the fatty tissues, the skin was sliced into a circle to match the circumference of the Franz cell. Franz diffusion cell apparatus has a 1.73 cm^2^ surface area with a 12 mL receptor volume capacity. Phosphate buffer solution at pH 5.5 was supplied to the receptor compartment of the Franz cell apparatus to serve as the simulated skin media. The Franz cell maintained a temperature of 37 °C with regular stirring. About 1 g of each gel was placed on the rat’s skin and in the donor compartment. Frequent samples from the receptor compartment were taken out at 0, 0.25, 0.5, 1, 2, 3, 4, 5, 6, 12, and 24 h, followed by an immediate replacement with an equal volume of PBS to maintain sink conditions. Samples were examined at λ_max_ = 272 nm using a UV-vis spectrophotometer (Bio-Tek Instruments, Bad Friedrichshall, Germany) to determine the total amount of permeated drug. The resulting data about drug permeability for MNTG and MNPG were plotted versus time [34]. The same methodology was carried out at pH 7.4 (blood pH) to ascertain the effects of pH on permeability. 

### 2.10. In Vitro Antifungal Activity

The cup plate technique was used to assess the antifungal properties of the developed MNTG (transethosomal gel), MNPG (plain gel), and marketed cream (Daktarin^®^ cream 2%) against the fungal strain *Candida albicans* (ATCC 10231) [35]. Fluconazole was used as a standard drug. Sabouraud dextrose agar medium was measured and dissolved in water in the desired proportion. The nutrient medium was sterilized for 15 min at 121 °C in an autoclave vacuum. In an aseptic environment, the nutrient medium was taken from the autoclave and cooled to 60 °C. The melted nutrient medium was poured into a Petri dish, and the tested microorganism was added and mixed to inoculate the medium. A sterile borer was used to create a well when the plate was allowed to solidify. Each well received the studied samples kept aside for an hour. An incubator was used to incubate the plates at 37 °C. After 48 h, the zone of inhibition (ZOI) was determined in millimeters by employing a simple graduated scale.

### 2.11. In Vivo Skin Irritation Study

Wistar albino rats weighing 110–130 g were used for the experiment for the in vivo skin irritation study. The Pharmacy Animal Ethics Committee at the Faculty of Pharmacy, IUB, Pakistan, approved the handling procedures under registration number PAEC/23/94. The methodologies were adapted to the UK Animals (Scientific Procedures) Act 1986 ethics and guidelines. Following the Organization for Economic Co-operation and Development (OECD) test guidelines 404, this study was executed for acute cutaneous irritation and corrosion. Under typical laboratory conditions, animals were accommodated appropriately in polycarbonate cages with free access to food and water. The animals were separated into three groups of six (n), and each group was caged separately [36].

Group I: Animals received the treatment with MNPG (Plain Gel).

Group II: The animals received the treatment with optimized formulation MNTG (transethosomal gel).

Group III: Animals received the treatment with marketed cream (Daktarin^®^ cream 2%).

Before the study began, rat hairs were removed from the experimental site using a hair-removal cream. Approximately 0.5 g of each tested formulation was topically applied to the skin surface for each group of rats. The tested formulations were removed after 24 h, and the skin surface was cleansed using distilled water. After that, the experimental sites were checked for skin irritation at 0 h, 24 h, and 48 h [37,38]. The primary cutaneous irritancy index was assessed by combining the results for each group’s erythema and edema scores. For each animal, the observations were documented as scores in numbers [39]. Depending on the level of erythema/edema, the mean erythema/edema scores were recorded: absence of erythema/edema = 0, very slight erythema/edema = 1, slight erythema/edema = 2, moderate erythema/edema = 3, severe erythema/edema = 4.

The MNTG (MT-8) scores were then contrasted with those of MNPG and marketed cream.

### 2.12. Statistical Analysis

The study’s findings were statistically and scientifically examined using Graph Pad Prism version 8.4.3 and IBM SPSS Statistics version 23. Different statistical tools, such as paired sample *t*-tests and ANOVA (analysis of variance), were applied for the interpretation of data. For the results to be deemed statistically significant, the *p*-values had to be less than 5% (*p* < 0.05).

## 3. Results and Discussion

### 3.1. In Vitro Characterization of MCNR-Loaded Transethosomes 

#### 3.1.1. Entrapment Efficiency

Table 2 demonstrates the %EE of the prepared formulations where the range of entrapment efficiency for all the transethosomal suspensions was 74.45 ± 1.15% to 89.93 ± 1.32%. The maximum %EE (89.93 ± 1.32%) was exhibited by formulation MT-8, containing 85% lecithin and 40% ethanol. It can be ascribed to the concentrations of lipid and ethanol, where %EE is strongly correlated with these two substances. %EE increases along with particle size at higher lipid concentrations [29]. Previous studies have demonstrated that a rise in ethanol content reduces vesicular size and increases entrapment efficiency, but only to some extent because greater ethanol concentrations cause leaky vesicles with lower %EE even at the same level of lipid content [40]. Moreover, the ability of transethosomes to entrap hydrophobic MCNR may be attributed to the electrostatic interaction between the positively charged drug molecule and the negatively charged transethosomes [41]. Ultimately, the high %EE of MCNR drug is also because of its great lipophilic nature, as all of the added drug quantity stays intact inside the lipophilic core [42]. Moreover, the drug-entrapment efficiencies of the formulations is increased by the edge activators utilized in transethosomal formulations, which can also help hydrophobic drugs become soluble [43]. Because of their outstanding colloidal stability and high entrapment efficiencies, transethosomes were investigated for efficient transdermal delivery of various bioactive compounds in a previously reported study [44].

#### 3.1.2. Determination of Particle Size

Vesicular sizes ranged from 139.3 ± 1.14 nm to 380.8 ± 1.21 nm for all the MCNR-TE formulations, as represented in Table 2. Among all formulations, MT-8 displayed the smallest particle size (139.3 ± 1.14 nm). Drug distribution, diffusion, release mechanisms, and skin depot are significantly impacted by particle size [45]. The particle size and the quantity of drug loaded in nanoparticles are impacted by several parameters, including chemical structure, amount and nature of drug and lipid used, and experimental methodologies [40]. A direct correlation between particle size and lipid concentration was found in the study results, as shown in Table 2. It was found that raising the concentration of lipid content in the formulation caused the particle size to grow larger. All formulations had an inverse correlation between the mean particle size and an upsurge in ethanol content (up to 40%). Formulations having 40% ethanol concentration displayed smaller particle sizes than those with 30% and 20% ethanol content. Following earlier reported findings, a rise in ethanol concentration lowers the size of the particles due to the ethanol hydrocarbon chain’s interpenetration, which reduces the size of the particles by lowering the thickness of the vesicular membrane [46]. Additionally, it was discovered that by reducing the lecithin-oleic acid ratio, the size of the vesicles was decreased. This is due to the fact that oleic acid serves as a surfactant, stabilizing the particles and preventing coalescence or growing too large, and hence causes a reduction in the size of the vesicles. Larger particle sizes may result from a rise in the lecithin: oleic acid ratio. This may be due to less surfactant, which would otherwise stabilize the particles and permit them to aggregate or coalesce [5]. 

Moreover, the sonication process significantly impacts vesicle size [34]. As a result, formulation MT-8 was seen as being optimized because it had the smallest particle size, which may be related to the proper process variables.

#### 3.1.3. Determination of PDI

The PDI of a formulation is a significant indicator in characterizing the homogeneity of nanoparticles in the dispersed system. The PDI of all developed MCNR-TE formulations ranged from 0.188 ± 0.05 to 0.426 ± 0.02, as shown in Table 2. The formulation MT-8, with the lowest PDI of 0.188 ± 0.5, indicates the establishment of a homogeneous transethosomal suspension. When the PDI is less than 0.5, the system is monodisperse, which satisfies the prerequisites for drug delivery. Khan et al. (2019) [47] developed rifampicin-loaded niosomes and found similar outcomes. It is clear that vesicles with a PDI less than 0.5 are deemed minimal, or show no aggregation, and their low polydispersity indicates a stable formulation system [47]. PDI values indicate a restricted size distribution and homogeneous dispersion between 0.1 and 0.5 [48]. Vast size distribution is demonstrated by a PDI value greater than 0.7. Lipids and edge activators are vital ingredients in transethosomal formulations that can have an impact on the PDI. Transethosomes with higher lipid concentrations often have larger vesicles. A wider size distribution and a greater PDI may follow from this [49]. However, a higher edge activator concentration can result in smaller, more homogeneous vesicles and a lower PDI. The increased edge activator concentration was associated with a small polydispersity index (PDI) [50]. Thus, a higher lecithin: oleic acid ratio resulted in larger PDI, while a lower lecithin: oleic acid ratio resulted in smaller PDI and more uniform vesicles. 

#### 3.1.4. Determination of Zeta Potential

A crucial factor in the stability of a colloidal dispersing system is the zeta potential [51], which demonstrated the charge’s electrostatic stability on the drug nanoparticles. The zeta potential of all the MCNR-TEs formulations ranged from −14.5 ± 0.27 to −28.2 ± 0.52 mV, as represented in Table 2. The optimized formulation, MT-8, had a zeta potential value of −18.1 ± 0.10 mV, which is attributed to the negative charge of the phospholipids. In addition, the presence of ethanol, which gives the polar head groups of the phospholipids a negative charge, was thought to contribute to the cause of the negative charge. Despite the low values of zeta potential, the stability of the prepared vesicles is assigned to the presence of ethanol that modifies the net charge of the systems into some degree of steric stabilization; this would reduce the aggregation of ethosomal vesicles and hence enhance the stability of these nanocarriers [52]. The net negative charge produced by ethanol in the vesicular system significantly contributes to improve the stability and penetration of transethosomes [53]. High ethanol concentrations, typically between 20% and 40%, have been associated with high zeta potentials, which delay the formation of agglomerates by electrostatic repulsion and increase vesicle stability [54]. Interestingly, vesicles may precipitate and become unstable at ethanol concentrations lower and higher than those mentioned above.

### 3.2. Optimum Formula Selection

There were nine formulations developed in total, but MT-8 was selected as the optimal formulation by considering the evaluation parameters because it exhibited the maximum entrapment efficiency (89.93% ± 1.32%), and the lowest particle size (139.3 ± 1.14 nm) and PDI (0.188 ± 0.05), and a good zeta potential (−18.1 ± 0.10 mV). The optimum formula MT-8 was comprised of 40% ethanol and a lecithin: oleic acid ratio of 85:15. The observed outcomes of MT-8 are displayed in Table 2 to confirm the experiment’s validity. MT-8 was selected for further research studies because it exhibited higher entrapment efficiency and the lowest particle size and PDI, as well as a suitable zeta potential.

### 3.3. In Vitro Characterization of Optimum MCNR Formulation

#### 3.3.1. Scanning Electron Microscopy (SEM)

**Figure 1:**
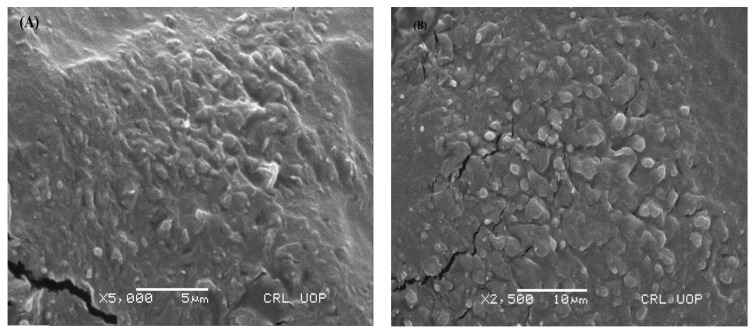
SEM images of optimized MT-8 formulation at 5 µm (**A**) and 10 µm (**B**).

SEM analysis was done at resolutions of 5 and 10 µm to study the surface morphology of the optimized MT-8 formulation, as illustrated in Figure 1. An SEM is a form of electron microscope that examines a sample with a concentrated beam of electrons to create photographs [55]. SEM images of the developed transethosomes represented almost spherical, homogeneous, and uni-lamellar nano-vesicles. The vesicle surface was smooth. Spherical-shaped vesicles predominated. Figure 1 depicts the active drug loaded in transethosomes as distributed white particles under high magnification across the network. The combination of 40% ethanol and 85:15 lipid/edge activator with increased and high encapsulated drug concentration resulted in spherical-shaped transethosomes with a smooth surface.

#### 3.3.2. Fourier-Transform Infrared Spectroscopy (FT-IR) Analysis

**Figure 2:**
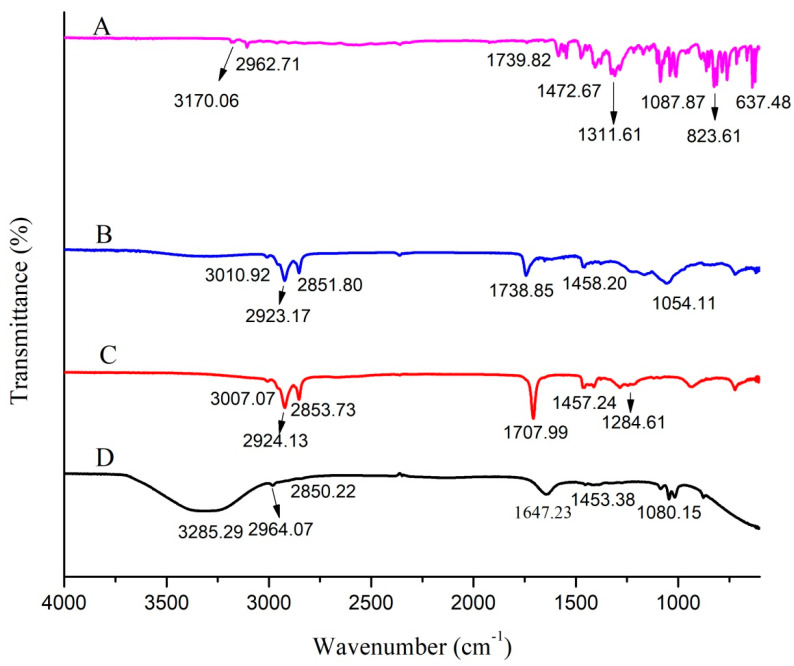
FTIR spectra of MCNR (**A**), lecithin (**B**), oleic acid (**C**), and optimized formulation MT-8 (**D**).

Identifying the drug–excipient interaction has been made possible with the help of IR spectrophotometry. FTIR analyses were carried out on the pure MCNR, phospholipid (lecithin), oleic acid, and the optimized formulation MCNR-TEs (MT-8) in the range of 500–4000 cm^−1^, as demonstrated in Figure 2. The FTIR spectrum of MCNR was characterized by bands at 3170.06 cm^−1^ (–C–N stretch), 3108.34 (–C–H stretch) aromatic, 2962.71 cm^−1^ (–C–H stretch) aliphatic, 1739.82 cm^−1^ (C=O stretch), 1546.93 cm^−1^ (–C–C bending), 1472.67 cm^−1^ (–CH_2_ stretch), 1311.61 cm^−1^ (–C–N stretching), 1087.87 cm^−1^ (–C–O stretch), 823.61 cm^−1^ (aromatic bend), and 637.48 cm^−1^ (–C–Cl stretch). The phospholipid’s FTIR spectra peaked at 1458.20 cm^−1^, corresponding to the –CH_2_ bend. At 3010.92 cm^−1^, the peak represented OH stretch. The C–H bands of phospholipid were found at 2923.17 cm^−1^ and 2851.80 cm^−1^. The spectra showed phospholipid’s carbonyl group (C=O) at 1738.85 cm^−1^. The peak at 1054.11 cm^−1^ showed a C–O stretch of phospholipid. 

The FTIR spectra of oleic acid displayed OH stretching at 3007.07 cm^−1^. Due to the C–H bands, oleic acid showed two distinct absorbance peaks at 2924.13 cm^−1^ and 2853.73 cm^−1^. Maximum absorbance of oleic acid was recorded at 1707.99 cm^−1^, indicating C=O stretch. The peak at 1457.24 cm^−1^ was associated with the CH_2_ stretch. The peak at 1284.61 cm^−1^ indicated –C–O stretch. A new major broad stretch that occurred at 3000–3500 cm^−1^ corresponding to the O–H group was visible in the spectra of optimized formulation (MT-8). C–H bands for MT-8 were noted at 2964.07 and 2850.22 cm^−1^. The C=C stretch of benzene or phenyl in MT-8 was observed at 1647.23 cm^−1^. The MT-8 peaked at 1453.38 cm^−1^, recognized as a –CH_2_ bend. The peak at 1080.15 cm^−1^ showed a C–O stretch in MT-8. FTIR spectra of the optimized formulation (having MCNR drug, phospholipid, and oleic acid) compared with their individual FTIR spectrum exhibited the presence of all chief distinctive peaks without any noticeable shifts, demonstrating the absence of any interactions. The outcomes demonstrated that all characteristic peaks seen in the MT-8 formulation were identical to those shown for MCNR alone, revealing that the drug and excipients are compatible and the drug is stable when excipients are present.

#### 3.3.3. Differential Scanning Calorimetry (DSC) Analysis

**Figure 3:**
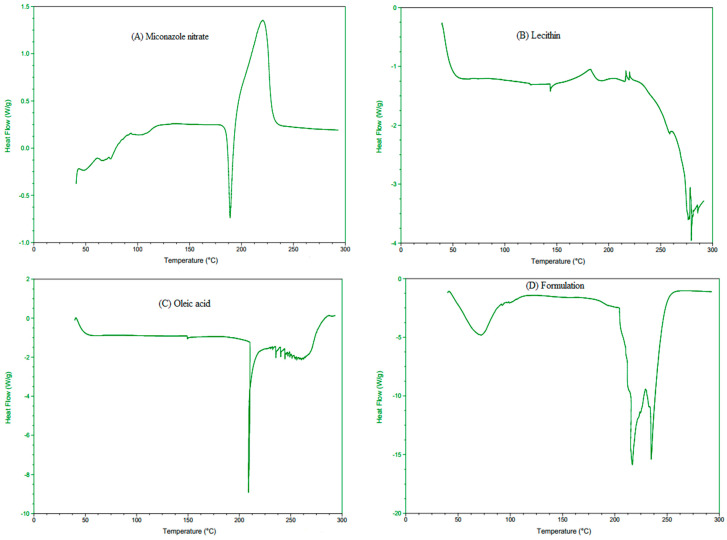
DSC thermograms of MCNR (**A**), lecithin (**B**), oleic acid (**C**), and optimized formulation (**D**).

DSC is a calorimetric method for determining a drug’s solubility and physical state in lipid vesicles. The DSC thermograms of the pure MCNR (drug), lecithin, oleic acid, and optimized transethosomal formulation (MT-8) were studied, as depicted in Figure 3. The DSC thermogram (A) of pure MCNR exhibited a sharp endothermic peak at 186.48 °C, which indicated the melting point of MCNR [3]. Thermogram (D) showed a less pronounced peak at 72.09 °C, indicating that MCNR was effectively entrapped in transethosomes [56]. These findings support earlier research by Bodade et al. (2013) [56] and Verma et al. (2014) [57]. Bodade et al. (2013) [56] noted an identical thermal response in repaglinide-loaded ethosomes for transdermal delivery through DSC analysis, and Verma et al. (2014) [57] demonstrated that clotrimazole was entrapped in oleic acid vesicles. These findings showed that phospholipid-based nano-carriers with ethanol and unsaturated fatty acid content can successfully encapsulate a range of drug molecules. The lecithin thermogram (B) displayed an endothermic peak at 279.55 °C, and the oleic acid thermogram (C) exhibited a large endothermic peak at 208.89 °C. The MT-8 formulation thermogram (D) showed a slight shift in the oleic acid peak to 216.55 °C and a lecithin prominent thermo-tropic transition peak to 234.86 °C. The MCNR, lecithin, and oleic acid created a bilayer when combined in formulation, lowering their crystallinity. The absence of melting endotherm of MCNR in the thermogram of MT-8 represented the existence of the drug in an amorphous state that is more soluble. The suppression of MCRN crystallization and solubilization in transethosomes may be the reason for the shift in melting behavior. This led to the conclusion that the MCNR (drug) was in an amorphous condition in the developed transethosomes. An increase in solubility results from a drug’s physical state changing to an amorphous or partially amorphous state, which induces a high disorder and high-energy state [58].

### 3.4. Stability Studies of Optimized Transethosomes Formulation 

The most appropriate formulation (MT-8) was selected for further investigations based on the findings represented in Table 3 and subjected to stability analysis for further 90 days in order to determine any variation in pH, color, %EE, and particle size at 4 °C and 25 °C by comparing the initial results with the measurements taken after 45 and 90 days. The findings showed that formulation MT-8 was highly stable at a low temperature of 4 °C without showing any considerable change in color, pH, %EE, or particle size. However, some variations were noted in these parameters at 25 °C temperature. A slight color shift from milky white to light yellow was seen in transethosomal suspension after 90 days at room temperature (25 °C), which may have been caused by the oxidation of lipids or extracts at higher temperatures. No color change was seen at low temperatures (4 °C). The pH slightly changed with time, and more pronounced changes in pH were observed at 25 °C. The system becomes more viscous when ethanol is present. However, a lipid bilayer’s gel-to-liquid shift could occur at higher temperatures, leading to fluidization or leaky vesicles with lowest %EE, pH, and larger vesicle size [34,40]. This demonstrates that transethosomes kept their stability and integrity at 4 °C. Therefore, in order to prevent any loss of drug and other instability issues, it is advised that the prepared transethosomes should be kept in a refrigerator (4 °C) for further use.

### 3.5. Evaluation of Gels

#### 3.5.1. Organoleptic Investigation and Stability Studies

Thermostability and organoleptic properties of the optimized MNTG (MT-8) and MNPG (plain) gels were assessed, as demonstrated in Table 4. Stability of both formulations was investigated for 3 months (90 days) by exposing them to a range of temperatures (8 °C, 25 °C, 40 °C, 40 °C ± 75% RH). When the organoleptic characteristics of gels were visually examined, it appeared white and had an adequate homogeneous texture. None of the formulations changed color. Minor variations were noticed, as shown in Table 4. Initially, MNTG and MNPG were stable at 8 °C and 25 °C for the entire investigational period.

Additionally, we noted that MNTG began to turn off-white at the end of the second month at higher temperatures (40 °C and 40 °C ± 75% RH) and turned light yellow from the third month. This could be explained by the lipid and transethosomes instability at elevated temperatures and humidity. During the first two months, smoothness remained constant, but by the third month, the MNTG and MNPG formulations at high temperatures had begun to thin, due to which their viscosity decreased. Throughout the investigational period, neither liquefaction nor odor change was observed in MNTG or MNPG. Similarly, no variation in appearance was noticed, as reported in Table 4.

#### 3.5.2. Analysis of pH

**Figure 4:**
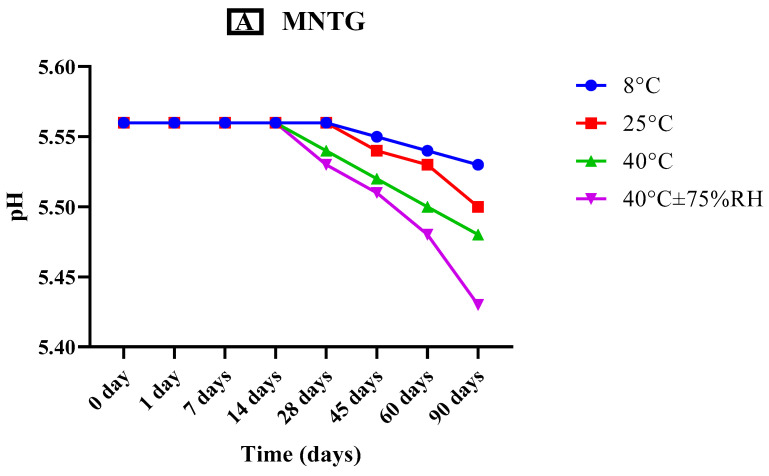

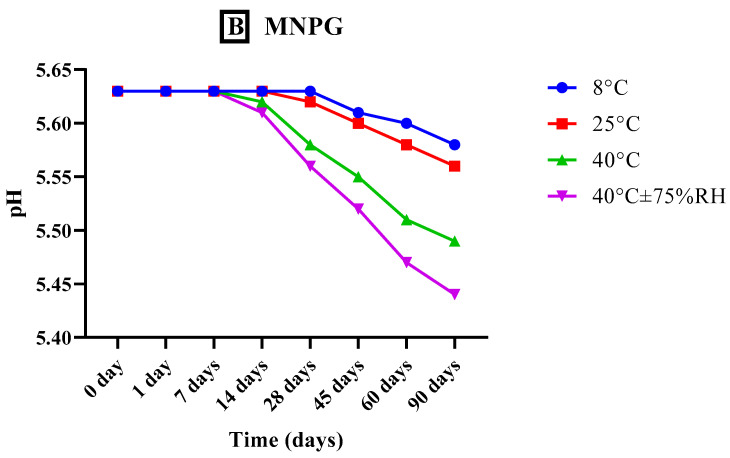
pH of MNTG (**A**) and MNPG (**B**) at various storage temperatures for 90 days. Two-way ANOVA and paired sample *t*-test at confidence interval of 95% showed significant differences (*p* < 0.0001) at different storage temperatures.

pH is a crucial factor in determining the durability and efficiency of skin treatments. According to various publications, the skin has a variable pH range, ranging from approximately 4.0 to 7.0. Normally, it tends to lie between 4.5 and 6.0. However, 5.5 is thought to be the skin’s natural pH. Thus, the topically applied optimal formulation should be closer to this pH limit. [29]. The pH of the MNTG (5.56 ± 0.13) and MNPG (5.63 ± 0.21) both showed a slight fall in pH over 90 days, which is represented graphically by Figure 4. This fall was more constant at lower temperatures, 8 °C and 25 °C. This indicates that gels are more stable at lower temperatures while the whole pH shift is within safe limits, supporting their usage topically. In earlier research, Abdulbaqi et al. (2018) developed a transethosomal gel for colchicine delivery through the skin and reported similar outcomes [59]. However, MNTG exhibited a considerable fall in pH with the passage of time especially at higher temperatures of 40 °C and 40 °C ± 75% RH, which may be caused by the active drug leaking out, disruption to the lipid bilayer of suspended transethosomes, and an increase in fluidity at higher temperatures [60]. Thus there is a noticeable reduction in pH of MNTG after 3 months at higher temperatures of 40 °C and 40 °C ± 75% RH. Therefore, the transethosomal gel is stable when stored at both room temperature and in the refrigerator.

Both MNTG and MNPG (gels) showed significant differences (*p* < 0.0001) in pH at various times and temperatures by applying ANOVA at the confidence interval of 95%. Furthermore, after the execution of the paired sample *t*-test for a comparative study of both MNTG and MNPG gel samples under all storage situations, significant changes were noted in pH.

#### 3.5.3. Measurement of Conductivity

**Figure 5:**
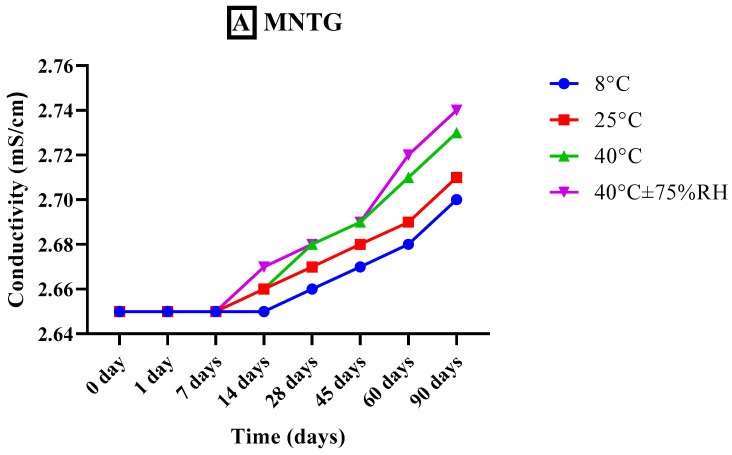

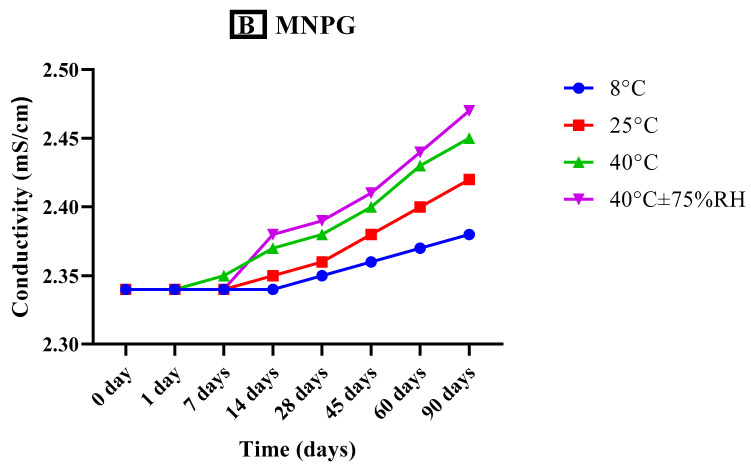
Conductivity of MNTG (**A**) and MNPG (**B**) at various storage temperatures for 90 days. Two-way ANOVA and paired sample *t*-test at confidence interval of 95% showed significant differences (*p* < 0.0016) at different storage temperatures.

Conductivity provides information regarding stability by analyzing the free ions and water present in the system. The optimized MNTG (MT-8) and MNPG (plain gel) were observed for conductivity at different storage temperatures, i.e., 8 °C, 25 °C, 40 °C, and 40 °C ± 75% RH for 90 days, as represented graphically in Figure 5. Newly developed plain and transethosomal gels have 2.34 mS/cm and 2.65 mS/cm electrical conductivities, respectively, but freshly prepared MNTG conductivity was significantly higher than that of MNPG. However, their conductivity had increased slightly after 90 days, especially at elevated temperatures, which might be associated with larger globules and convergence of multiple system phases [61].

Additionally, previous research demonstrated that drug-loaded formulations always have higher conductivity than drug-free formulations [62]. Both MNTG and MNPG (gels) showed significant differences (*p* < 0.0016) in conductivity at various times and temperatures by applying ANOVA at the confidence interval of 95%. Furthermore, after the execution of the paired sample *t*-test for a comparative study of both MNTG and MNPG gel samples under all storage situations, significant changes were noted in conductivity.

#### 3.5.4. Viscosity Analysis

**Figure 6:**
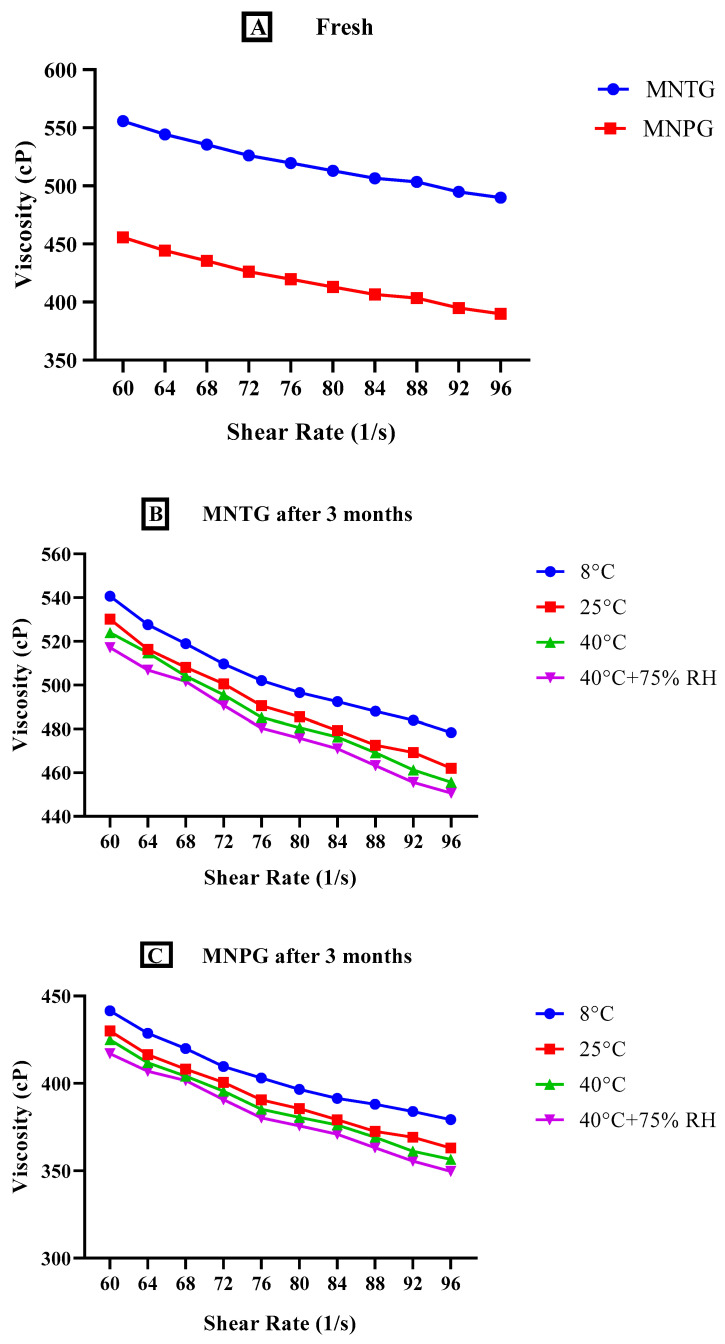
Viscosity analysis of freshly prepared MNTG and MNPG at 25 °C (room temperature) (**A**), optimized MNTG after 3 months (**B**), and MNPG after 3 months (**C**), at various storage temperatures (8 °C, 25 °C, 40 °C, 40 °C ± 75% RH). Two-way ANOVA and paired sample *t*-test at confidence interval of 95% showed significant differences (*p* < 0.0001) at different storage temperatures.

The viscosity of a gel, which demonstrates the physical stability of the formulation aimed for topical use, provides a good description of its flow characteristics [29]. The viscosity record of both freshly developed optimized MNTG (MT-8) and MNPG (plain gel) was observed at different storage temperatures, i.e., 8 °C, 25 °C, 40 °C, and 40 °C ± 75% RH, for 3 months (90 days). The variations in the viscosity of freshly prepared optimized transethosomal gel (MNTG) and plain gel (MNPG) at room temperature and after 3 months at various temperatures are depicted in Figure 6. The viscosity of gels gradually dropped as time passed.

Moreover, more decline in viscosity was observed at higher temperature ranges. The nanosized particles of transethosomal suspension seem to be stationary, but in fact they are moving slowly, so when the shear rate rises they attempt to line up themselves and can effortlessly pass and recover by Brownian movement, which may be responsible for this variation over a limited range of shear rate (60–96 s^−1^) [63]. As a result, a non-Newtonian pseudo-plastic behavioral pattern was noticed. The findings were further supported by Yadav’s earlier investigations [64], which showed that transethosomal gel had better performance than plain gel due to its robust gel matrix. Both MNTG (MT-8) and MNPG (gels) showed significant differences (*p* < 0.0001) in viscosity at various times and storage temperatures by applying ANOVA at the confidence interval of 95%. Furthermore, after the execution of the paired sample *t*-test for a comparative study of both MNTG and MNPG gel samples under all storage situations, significant changes were noted in viscosity.

#### 3.5.5. Spreadability

**Figure 7:**
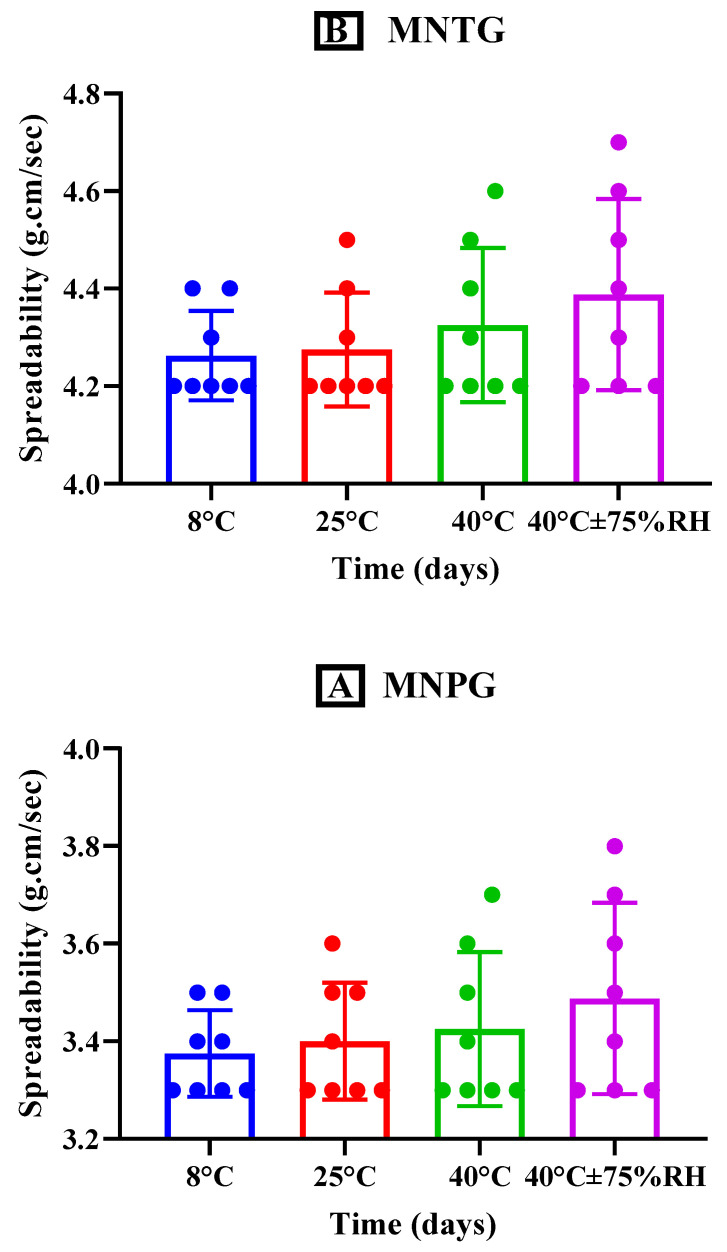
Spreadability of MNTG (**A**) and MNPG (**B**) at various storage temperatures for a time interval of 90 days. Two-way ANOVA and paired sample *t*-test at confidence interval of 95% showed significant differences (*p* < 0.0001) at different storage temperatures.

The spreadability of gel is an essential factor influencing how evenly the formulation is applied to the skin. The spreadability of a gel affects its effectiveness since a high spreadability indicates a short time of spreading, which improves patients’ compliance. The spreadability of the plain and optimized transethosomal gels stored at several temperatures (as previously mentioned) for the time interval of 90 days is depicted in Figure 7, which demonstrates a slight increase in spreadability resulting from the reduction in viscosity when a specific force is exerted over a particular amount of time [65]. A significant increment in spreadability was seen for both MNTG and MNPG (gels) at different temperatures and times at the 95% confidence interval (CI) using ANOVA. Additionally, after doing a paired sample *t*-test intended for multiple comparative studies of both MNTG and MNPG gels under all storage circumstances, significant differences (*p* < 0.0001) were also found.

### 3.6. In Vitro Release Study

**Figure 8:**
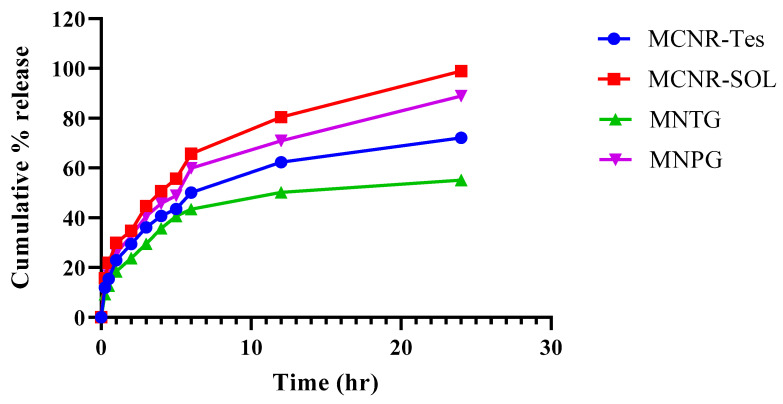
In vitro drug release pattern of MCNR solution (MCNR-SOL), MCNR transethosomal formulation (MT-8), optimized transethosomal gel (MNTG), and MCNR plain gel (MNPG) at pH 5.5 (human skin pH) for a time duration of 24 h.

In vitro analyses of the release patterns of MCNR solution (MCNR-SOL), MCNR transethosomal formulation (MT-8), optimized formulation (MT-8)-loaded transethosomal gel (MNTG), and MCNR solution-loaded plain gel (MNPG) were conducted at pH 5.5. Figure 8 displays the results. It was seen that 62.36% of the MCNR was released from MT-8, while about 80.44% of the MCNR was released from MCNR-SOL within the first 12 h. In contrast, MNTG showed controlled release, and only about 50.23% MCNR was released in the first 12 h, compared to MNPG gel from which about 70.98% MCNR was released within the first 12 h. MNPG release rate was comparatively more significant than the release of MCNR from MNTG gel. A maximum of 72.13% and about 55.16% of drug release was observed from MT-8 and MNTG gel, respectively, in 24 h, whereas MCNR-SOL released nearly all of the drug, around 98.99%, and MNPG gel released 88.95% of the drug in 24 h, as shown in Figure 8. According to the observations, MNTG gel exhibited a controlled release of MCNR because only 55.16% of the drug was released within the first 24 h. 

The study’s findings showed that MT-8 initially released the drug in a burst manner, probably due to drug desorption and release from the vesicle surface, and then slowly released, indicating a controlled release pattern. This demonstrated that the developed transethosomes could sustain drug release for a long time, as reported earlier [66]. According to Verma and Utreja (2018) [44], transethosomes sustained the release of econazole nitrate for about 24 h. This study demonstrated that transethosomes, because of their distinctive behavior of sustained release, can be investigated for a drop in the dosing frequency of commonly administered drugs. Additionally, a lower release rate was seen with the MNTG gel than the MT-8 because MCNR must first be released from the transethosomes before it can be diffused into the matrix of the carbopol gel. 

#### Kinetic Models for Drug Release Characterization

The in vitro release pattern of MCNR from MT-8 and MNTG gel was investigated using various kinetic models. Table 5 presents the R^2^ values for each employed model. Korsmeyer–Peppas models had the highest R^2^ values for in vitro drug release of MT-8 and MNTG gel, and as a result, this model was determined to be the best fit. The values of “n’’ for MCNR in MT-8 and MNTG-gel were found to be 0.353 and 0.338, respectively, displaying release following the Fickian diffusion model. It demonstrated that the release of MCNR from optimized formulation (MT-8) and gel (MNTG) was diffusion-controlled, as exhibited in Table 5.

### 3.7. Ex Vivo Permeation Study

**Figure 9:**
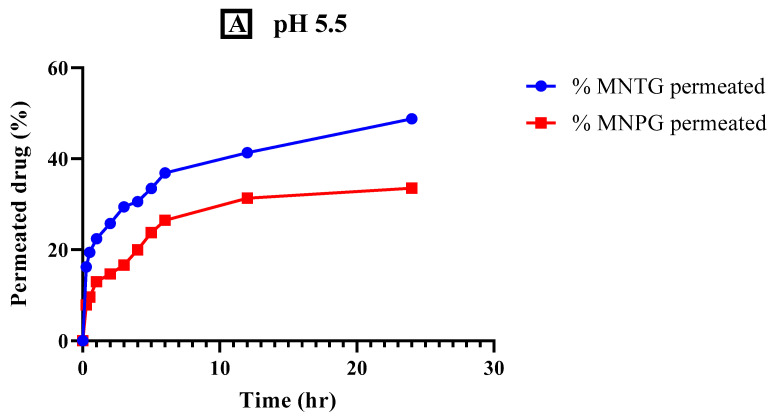

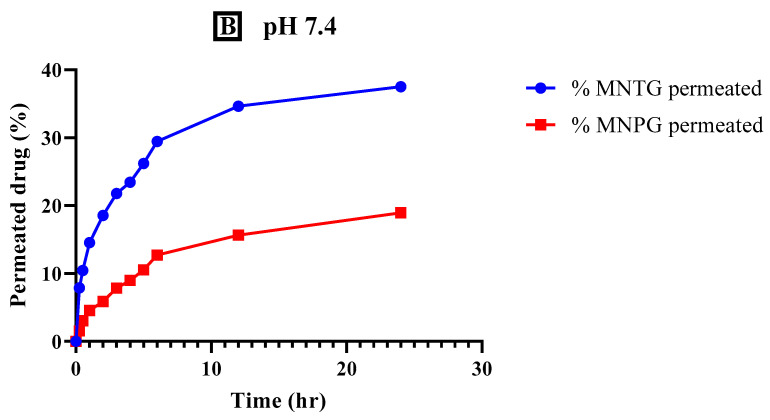
% drug permeation comparison of optimized transethosomal gel (MNTG) and MCNR plain gel (MNPG) at pH 5.5 (human skin-like) (**A**) and at pH 7.4 (human blood-like) (**B**) for a time duration of 24 h. Significant outcomes were obtained (*p* < 0.0001).

In transdermal drug delivery systems, permeation is a crucial factor. Figure 9 displays ex vivo permeation analyses of optimized MNTG (transethosomal gel) and MNPG (plain gel) formulations at pH 5.5 (human skin-like) and pH 7.4 (human blood-like). As shown in Figure 9, it was observed that the permeation of MCNR from MNTG at pH 5.5 was 48.76%, which was significantly greater (*p* < 0.0001) than the permeation of MNPG (33.52%). The permeation of MCNR from MNTG at pH 7.4 was 37.53%, which was significantly higher (*p* < 0.0001) than the permeation of MNPG (18.95%). The outcomes obtained under these two circumstances of pH similar to that of the human body showed that MNTG was more permeable at pH 5.5 than pH 7.4, demonstrating that the drug is retained more in the skin than in the bloodstream. Reduced drug systemic penetration may help to explain this. As a result, such formulation ought to be recommended for topical use instead of intravenous (IV) administration.

Table 6 displays the values for flux, enhancement ratio, and permeability coefficient, where the enhancement ratio showed that MCNR permeation from MNTG was raised by 1.50 compared to MNPG at pH 5.5. However, the enhancement ratio was only raised by 0.47 at pH 7.4. The adhesive and hydrophilic properties of the gelling agent used in this study, carbopol-934, as well as the suitable physical characteristics and efficient permeation of MCNR from MNTG via Fickian diffusion, all strongly suggest that MCNR-derived transethosomal gel can be used for topical application for local action [67]. Other research studies with similar findings showed that the transethosomal gel had higher permeability than the plain gel. The characteristics of the phospholipid used to develop transethosomes and their nanosized vesicles are more likely to cause the enhanced permeability of transethosomes [52]. A further explanation could be the presence of ethanol and oleic acid in the transethosomal gel, which acts as a penetration enhancer [68].

### 3.8. In Vitro Antifungal Activity

**Figure 10:**
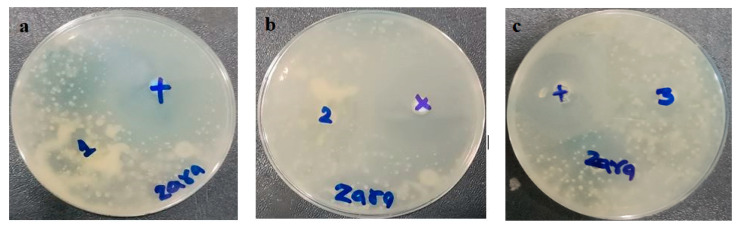
In vitro antifungal activity assessment of MCNR plain gel (MNPG), (**a**) prepared MCNR transethosomal gel (MNTG), (**b**) marketed product (Daktarin^®^ cream 2%), (**c**) exhibiting zone of inhibition against standard drug (Fluconazole).

The prepared MCNR transethosomal gel (MNTG), MCNR plain gel (MNPG), and marketed product (Daktarin^®^ cream 2%) were tested using the cup plate method for their antifungal activity against *Candida albicans*. The positive control used was fluconazole, which served as a standard drug. Findings are displayed in Figure 10 and Table 7. The zone of inhibition was measured to ascertain the antifungal activity [69]. It was found that the MNTG (transethosomal gel) had a 20.5 ± 0.02 mm zone of inhibition, while the marketed cream had a 16 ± 0.01 mm zone of inhibition. The least noticeable inhibitory zone of 11 ± 0.05 mm was measured in MNPG (plain gel). Results revealed that the MNTG (transethosomal gel) had more antifungal activity than the marketed cream and plain gel. Moreover, it was noticed that the antifungal activity of MCNR was enhanced by introducing it into transethosomal gel. This may be explained by transethosomes’ high degree of flexibility, which allowed them to penetrate the *Candida albicans* fungal cell walls, and by their suppression of ergosterol synthesis, which causes the lysis of fungal cell membrane and death [25]. The profound antifungal activity of MCNR obtained from the MNTG (transethosomal gel) was demonstrated in earlier research to be linked with nanosized vesicles, the mucoadhesive characteristics of the Carbopol, and the considerable solubility of MCNR upon the addition of surfactant.

### 3.9. In Vivo Skin Irritation Study

**Figure 11:**
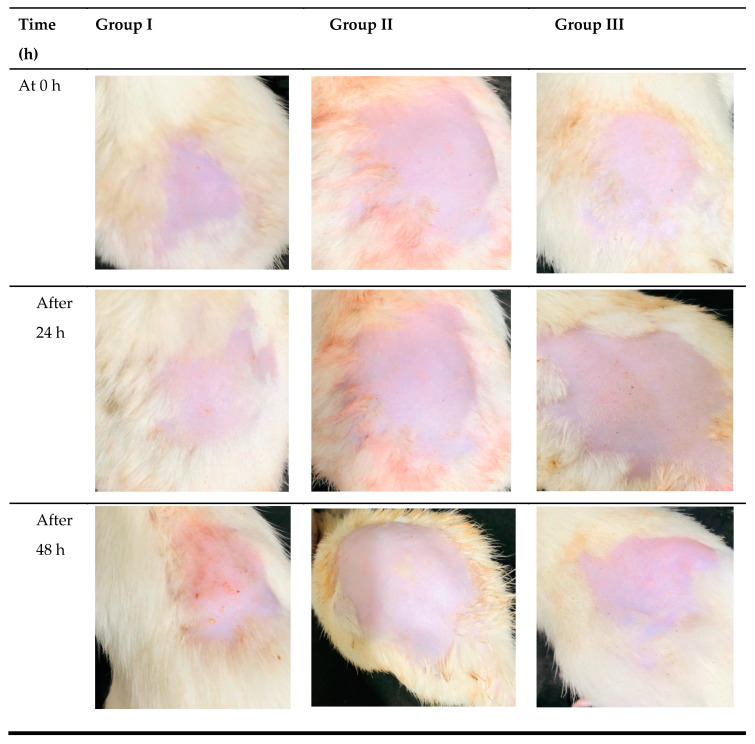
In vivo skin irritation test after MCNR plain gel (MNPG), (**Group I**), MCNR transethosomal gel (MNTG), (**Group II**), and marketed cream, (**Group III**).

Healthy albino rats were used for the skin irritation study, where the emergence of erythema and edema in the rats was noticed. Transethosomal gel preparation was favorable to rat skin according to the investigations on skin irritation. For 48 h, the rats in group II showed no severe symptoms of erythema and edema (Figure 11). After 48 h, the rats in group I showed moderate symptoms of erythema and severe edema. Rats in group III, however, displayed slight edema, as depicted in Figure 11. The primary cutaneous irritancy index score for MNPG (plain gel), MNTG (transethosomal gel), and marketed cream (Daktarin^®^ cream 2%) was investigated to be 2.9, 0.6, and 0.9, respectively. MNTG’s irritancy score was significantly lower (*p* ≤ 0.05) than the typical irritant’s. This might be because intact vesicles penetrate deeply, minimizing the direct interaction of MCNR molecules with the skin. The developed MCNR-transethosomal gel can be inferred to be less irritating, well-tolerated, and safe for application over the skin.

## 4. Conclusions

In the present study, transethosomes of miconazole nitrate were successfully developed and later incorporated in carbopol-934 gel. It demonstrated controlled drug release, making it appropriate for topical drug delivery. DSC and FTIR analyses exhibited high compatibility of the drug with the excipients in the formulation and showed its encapsulation within nanoparticles. The MNTG gel displayed three months of physical stability at different temperatures, and its pH stayed within the range of the skin, indicating that it will not irritate the skin. The transethosomal gel (MNTG) exhibited good spreadability, optimal conductivity, pH, and viscosity. In addition, when compared to the commercially available product (Daktarin^®^ cream 2%) and plain gel (MNPG), the formulation of miconazole nitrate as transethosomal gel has the capacity to overcome the barrier qualities of the skin and enhance the antifungal activity. Furthermore, preclinical investigations have revealed that the developed transethosomal gel (MNTG) is safe and non-irritant because it showed no clinical signs of erythema or edema. Thus, transethosomal gel loaded with miconazole nitrate could be an excellent product for treating cutaneous fungal infection. It can be inferred that the developed formulation MNTG may hold promise for the topical delivery of miconazole nitrate for treating different skin fungal infections with enhanced antifungal activity and may have significant advantages.

## Figures and Tables

**Table 1:** Composition of different MCNR-loaded transethosomal formulations.

Code	Drug (mg)	Lecithin: OA (mg)	Ethanol (*v*/*v*%)	PBS (pH 5.5) (*v*/*v*%)	Hydration Volume (mL)
MT-1	100	75:25	20	80	10
MT-2	100	85:15	20	80	10
MT-3	100	95:05	20	80	10
MT-4	100	75:25	30	70	10
MT-5	100	85:15	30	70	10
MT-6	100	95:05	30	70	10
MT-7	100	75:25	40	60	10
MT-8	100	85:15	40	60	10
MT-9	100	95:05	40	60	10

Abbreviations: OA, oleic acid; PBS, phosphate buffer solution; Note: All of the ingredients of the above-mentioned formulations were taken in 10 mL volume.

**Table 2:** Entrapment efficiency, particle size, polydispersity index, and zeta potential measurements of MCNR-loaded transethosomes (n = 3, mean ± standard deviation).

Code	Entrapment Efficiency (%EE)	Particle Size (nm)	Polydispersity Index (PDI)	Zeta Potential (mV)
MT-1	74.45 ± 1.15	343.7± 1.67	0.315 ± 0.01	−15.5 ± 0.21
MT-2	78.05 ± 0.34	376.5 ± 1.45	0.349 ± 0.06	−20. ± 0.14
MT-3	86.31 ± 2.17	380.8 ± 1.21	0.426 ± 0.02	−28.2 ± 0.52
MT-4	79.43 ± 1.67	202.5 ± 1.76	0.390 ± 0.04	−14.5 ± 0.27
MT-5	85.90 ± 2.17	288.4 ± 2.87	0.346 ± 0.08	−16.3 ± 0.37
MT-6	87.61 ± 1.74	310.0 ± 2.45	0.274 ± 0.09	−21.6 ± 0.62
MT-7	83.98 ± 1.12	174.6 ± 1.26	0.192 ± 0.03	−16.7 ± 0.14
MT-8	89.93 ± 1.32	139.3 ± 1.14	0.188 ± 0.05	−18.1 ± 0.10
MT-9	88.87 ± 2.42	196.0 ± 1.03	0.391 ± 0.07	−18.5 ± 0.65

**Table 3:** Stability of optimized transethosomes formulation (MT-8) at different storage temperatures (n = 3, mean ± standard deviation).

Parameter	Time (days)	4 °C	25 °C
pH	0	5.54 ± 0.13	5.54 ± 0.13
	45	5.52 ± 0.04	5.51 ± 0.24
	90	5.51 ± 0.11	5.47 ± 0.19
Color	0	Mw	Mw
	45	Mw	Mw
	90	Mw	Ly
% EE	0	89.93 ± 1.32%	89.93 ± 1.32%
	45	87.52 ± 0.14%	85.39 ± 1.25%
	90	84.79 ± 1.28%	80.14 ± 2.11%
Particle size (nm)	0	139.3 ± 1.14	139.3 ± 1.14
	45	1.43 ± 2.16	1.48 ± 1.47
	90	146.1 ± 0.05	159.4 ± 1.21

Abbreviations: Mw, milky white; Ly, Light yellow; EE, entrapment efficiency.

**Table 4:** Thermostability of MNTG (MT-8) and MNPG (plain gel) at different storage temperatures for a study period of 90 days.

Parameters	Temp.	Fresh		24 h		7 Days		14 Days		28 Days		45 Days		60 Days		90 Days	
		PG	TG	PG	TG	PG	TG	PG	TG	PG	TG	PG	TG	PG	TG	PG	TG
Color	8 °C	W_TE_	W_TE_	W_TE_	W_TE_	W_TE_	W_TE_	W_TE_	W_TE_	W_TE_	W_TE_	W_TE_	W_TE_	W_TE_	W_TE_	W_TE_	W_TE_
	25 °C	W_TE_	W_TE_	W_TE_	W_TE_	W_TE_	W_TE_	W_TE_	W_TE_	W_TE_	W_TE_	W_TE_	W_TE_	W_TE_	W_TE_	W_TE_	W_TE_
	40 °C	W_TE_	W_TE_	W_TE_	W_TE_	W_TE_	W_TE_	W_TE_	W_TE_	W_TE_	W_TE_	W_TE_	W_TE_	W_TE_	OWT	W_TE_	LY
	40 °C ± 75% RH	W_TE_	W_TE_	W_TE_	W_TE_	W_TE_	W_TE_	W_TE_	W_TE_	W_TE_	W_TE_	W_TE_	W_TE_	W_TE_	OWT	W_TE_	LY
Odor	8 °C	-	-	-	-	-	-	-	-	-	-	-	-	-	-	-	-
	25 °C	-	-	-	-	-	-	-	-	-	-	-	-	-	-	-	-
	40 °C	-	-	-	-	-	-	-	-	-	-	-	-	-	-	-	-
	40 °C ± 75% RH	-	-	-	-	-	-	-	-	-	-	-	-	-	-	-	-
Liquefaction	8 °C	-	-	-	-	-	-	-	-	-	-	-	-	-	-	-	-
	25 °C	-	-	-	-	-	-	-	-	-	-	-	-	-	-	-	-
	40 °C	-	-	-	-	-	-	-	-	-	-	-	-	-	-	-	-
	40 °C ± 75% RH	-	-	-	-	-	-	-	-	-	-	-	-	-	-	-	-
Appearance	8 °C	T_RP_	M_KY_	T_RP_	M_KY_	T_RP_	M_KY_	T_RP_	M_KY_	T_RP_	M_KY_	T_RP_	M_KY_	T_RP_	M_KY_	T_RP_	M_KY_
	25 °C	T_RP_	M_KY_	T_RP_	M_KY_	T_RP_	M_KY_	T_RP_	M_KY_	T_RP_	M_KY_	T_RP_	M_KY_	T_RP_	M_KY_	T_RP_	M_KY_
	40 °C	T_RP_	M_KY_	T_RP_	M_KY_	T_RP_	M_KY_	T_RP_	M_KY_	T_RP_	M_KY_	T_RP_	M_KY_	T_RP_	M_KY_	T_RP_	M_KY_
	40 °C ± 75% RH	T_RP_	M_KY_	T_RP_	M_KY_	T_RP_	M_KY_	T_RP_	M_KY_	T_RP_	M_KY_	T_RP_	M_KY_	T_RP_	M_KY_	T_RP_	M_KY_

Abbreviations: RH, relative humidity; PG, plain gel; TG, transethosomal gel; W_TE_, white; OWT, off-white; LY, light yellow; (-), no change; T_RP_, transparent; M_KY_, milky.

**Table 5:** Kinetic models applied on MCNR-TEs optimized formulation and MNTG (gel).

Formulation	Zero-Order	First Order	Higuchi	Korsmeyer–Peppas	
	R^2^	R^2^	R^2^	R^2^	N
MCNR-TEs	0.0170	0.7556	0.8917	0.9795	0.353
MNTG	−0.0907	0.5041	0.8470	0.9572	0.338

**Table 6:** Ex-vivo permeation assessment of different parameters for MNTG and MNPG.

pH 5.5			
Formulations	Flux (µg/cm^2^/h)	Permeability Coefficient (cm/h)	Enhancement Ratio
MNTG	26.01	0.0953	1.50
MNPG	17.34	0.0781	
pH 7.4			
MNTG	3.17	0.0319	0.47
MNPG	6.64	0.0112	

**Table 7:** In vitro antifungal activity of different samples against *Candida albicans*.

Sample	Named	Zone of Inhibition (mm)	
		* Positive Control	Test Samples
MNPG	1	30 ± 0.02 mm	11 ± 0.05 mm
MNTG	2	28 ± 0.03 mm	20.5 ± 0.02 mm
Marketed cream	3	30 ± 0.03 mm	16 ± 0.01 mm

* Positive Control used: fluconazole; MNPG, miconazole nitrate plain gel; MNTG, miconazole nitrate transethosomal gel.

## Data Availability

The corresponding author can provide the data described in this study upon request.
